# A Neck Mass of Thymic Origin in a Pediatric Patient

**DOI:** 10.7759/cureus.32468

**Published:** 2022-12-13

**Authors:** Audrey J Zauher, Jonathan Jacobs, Amal Isaiah

**Affiliations:** 1 Otorhinolaryngology - Head and Neck Surgery, University of Maryland School of Medicine, Baltimore, USA; 2 Pathology, University of Maryland Medical Center, Baltimore, USA; 3 Pediatrics, University of Maryland School of Medicine, Baltimore, USA; 4 Diagnostic Radiology and Nuclear Medicine, University of Maryland School of Medicine, Baltimore, USA

**Keywords:** hasall corpuscles, lateral neck mass, surgical excision, thymic mass, congenital neck mass

## Abstract

In this study, we present the case of a 10-year-old boy with a left-sided neck mass. Although most neck masses in children are non-cancerous, their etiology can be complex, especially in neck masses of congenital origin. The workup of a pediatric neck mass includes imaging and cytopathology. In this case, the histopathology of the excised mass revealed thymic tissue, which helped establish the diagnosis of a thymopharyngeal duct cyst. Thymophayngeal duct cysts, although rare, can be diagnosed preoperatively by characteristic tapering toward the mediastinum. Cytopathology may demonstrate Hassall corpuscles. These unique features can help disentangle the differential diagnoses, which commonly include thyroglossal duct cysts, venolymphatic malformations, and branchial cleft cysts.

## Introduction

The thymus develops in the ventral wings of the third pharyngeal arch and descends into the superior anterior mediastinum along the paired thymopharyngeal ducts typically during the seventh and eighth weeks of gestation [1]. The thymopharyngeal ducts run along the carotid sheaths from the angle of the mandible to the anterior mediastinum, deep into the thyroid gland and sternocleidomastoid muscles [2]. Following the descent, the ducts form epithelial cords that eventually involute and atrophy [3]. Thymic remnants can be found anywhere along the course of the thymopharyngeal ducts, either due to the sequestration of tissues during descent or the failure of the thymopharyngeal ducts to involute during development. In contrast, the thyroid originates between the foramen cecum near the base of the tongue and migrates inferiorly along a single midline thyroglossal duct, which eventually degenerates by the 10th week of gestation [4].

The most common congenital neck masses are thyroglossal duct cysts, branchial cleft cysts, and cystic lymphatic malformations [5]. Cervical thymic cysts are the rarest neck masses, ranging from branchial remnants to degenerating Hassall’s corpuscles [6]. One-half of these cysts extend into the mediastinum [7]. They can be distinguished by their location and whether they are cystic or solid. Cysts of thymic origin account for 0.3-2% of congenital neck masses [8-10]. Persistent thymopharyngeal duct cysts account for a little more than 7% of thymic remnants, whereas accessory cervical thymus and cervical thymic cyst variants constitute over 50% of thymic remnants [11]. Approximately 70% of these cysts occur in men [12]. Most lesions present as asymptomatic neck masses, although some rare complications, such as respiratory distress, have been reported [13,14]. Most thymic cysts present within the first decade of life and occur on the left side [5,10]. Among children with ectopic cervical thymic tissue, 97.5% have a normal mediastinal thymus [3,14].

In this study, we highlight the unique clinical, imaging, and histopathologic features of a thymopharyngeal duct cyst presenting as a lateral neck mass in a child. Our goal is to provide a standardized approach to their management aided by imaging, surgical intervention, and histopathologic pearls.

## Case presentation

A 10-year-old boy presented to the emergency department with a left-sided neck mass that was noticed about three weeks after an upper respiratory tract infection (URI). He denied dyspnea, dysphagia, chills, fever, otalgia, congestion, sore throat, and rhinorrhea on presentation. Upon examination, the mass, which extended from the angle of the left mandible to the left clavicle, was found to be soft, non-tender, mobile, and without associated cutaneous changes. Laboratory studies were largely unremarkable.

**Figure 1:**
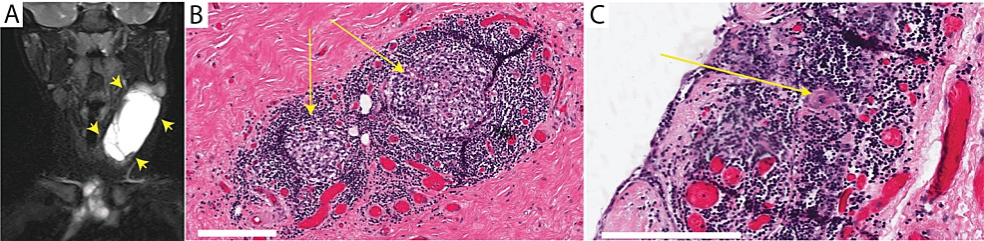
Imaging and histopathologic features of a neck mass in a child. T2-weighted magnetic resonance imaging of the neck in the coronal plane (A) demonstrates a large multiloculated cystic left-sided neck mass (arrowheads) that extends from C1/C2 level to the anterior mediastinal compartment. Histopathology of the excised mass at high power (B) shows a thymic cortex (arrows), filled with tightly packed thymocytes, and the less cellular medulla with epithelial cells is identified. Round keratinized formations consistent with Hassall corpuscles (C, arrow) are found in some of the islands (bar = 200 μm).

Computed tomography (CT) with intravenous contrast showed a large left-sided cystic mass extending from C1/C2 to the superior aspect of the anterior mediastinum, measuring 5 cm × 3 cm × 1 cm, located between the left sternocleidomastoid and the left carotid sheath and with some mass effects. Further investigation with magnetic resonance imaging (MRI, Figure 1A) showed septal enhancement within the mass.

During elective excision, the cyst capsule tapered inferiorly to a cord-like structure within the superior aspect of the anterior mediastinum. The mass was dissected off the carotid artery and sent for pathology. A gross examination of the excised mass revealed a pink-tan, smooth, and fibromembranous surface with fluid-filled subsurface cysts. Histopathologic examination of the cyst wall at low power revealed a flat epithelial lining with underlying fibrovascular tissue. At high power, the thymic cortex, filled with tightly packed thymocytes, and a less cellular medulla with epithelial cells were identified (Figure 1B), along with Hassall corpuscles (Figure 1C). These features contributed to the diagnosis of a thymopharyngeal duct cyst.

## Discussion

The typical workup of neck masses in children includes imaging (e.g., MRI) and possibly cytopathology following fine-needle aspiration. Some features may help distinguish thymic masses from other etiologies. An MRI can be used to confirm the presence of a mediastinal thymus and compare soft tissue densities between the suspect neck mass and the existing mediastinal thymus. Septations may be visible; however, thyropharyngeal duct cysts and thyroglossal duct cysts can be septated [15]. If further confirmation is warranted, fine-needle aspiration of the cyst may confirm the presence of thymic tissue. Complete surgical excision is recommended except in children without a mediastinal thymus.

Numerous similarities exist between the features of the neck mass in this case and those previously reported in the literature. Notably, the neck mass was asymptomatic, consistent with most other papers that describe thymopharyngeal duct cysts [3,6,8,12]. The neck mass was left-sided [3,7,12,13], and subsequent histopathology revealed Hassall corpuscles [3,7,8,12,13]. Central to the presentation of the mass, in this case, was its development within three weeks of a URI. Wagner et al. [7] observed this pattern; all seven cases that experienced a rapid increase in mass size had a recent URI.

The surgical excision of a suspected thymopharyngeal duct cyst was done via a lateral neck incision. Following a horizontal incision within a skin crease, a subplatysmal plane was developed and dissected to approach the cyst capsule. The mass was bluntly dissected using the concave surface of a dissecting hemostat to avoid puncturing the cyst. However, the fibrous capsule is typically tougher than the walls of other cystic lesions, such as lymphatic malformations. Once the anterior surface was freed, the posterior dissection was completed with a combination of a finger sweep and blunt dissection using a hemostat in a craniocaudal direction to avoid injury to the contents of the carotid sheath. A close relationship with the carotid sheath is specific to thymopharyngeal duct cysts. An extensive thymopharyngeal duct cyst that extends into the submandibular area and beyond places the facial nerve at risk, especially due to unusual branching patterns [16], and should be avoided by subplatysmal dissection and the possible use of neuromonitoring. The use of a drain and perioperative antibiotic prophylaxis is recommended. On histopathology, thymopharyngeal duct cysts demonstrated an epithelial lining, unlike other congenital neck masses. The presence of Hassall corpuscles, concentric islands of squamous cells with central keratinization, is pathognomonic for thymic lesions. Recurrence is rare following complete surgical excision. In this case, the surgery was completed without any complication, and the child did well postoperatively without any sign of recurrence at the two postoperative visits.

## Conclusions

Although neck masses in children can have various etiologies, identifying cord-like tapering associated with a lateral neck mass can suggest thymopharyngeal duct cysts and should be included in the differential diagnosis of neck masses in children. While excision and subsequent histopathology are confirmatory, a fine needle aspiration that reveals the thymic tissue is of high diagnostic yield in the setting of suggestive imaging features. Surgical excision is typically curative.
